# Medical student perceptions of reflective practice in the undergraduate curriculum

**DOI:** 10.12688/mep.19211.2

**Published:** 2022-10-27

**Authors:** Faye Gishen, Rima Chakrabarti

**Affiliations:** 1 University College London Medical School, London, WC1E 6DE, UK

**Keywords:** Reflection, reflective practice, medical students, professionalism, well-being

## Abstract

**Introduction: **Reflective practice (RP) forms a core component of medical professionalism but, despite its benefits, it remains largely undervalued among medical students. The aim of this study was to explore medical students’ attitudes and barriers to engagement with RP in the undergraduate programme at a UK based medical school.

**Methods: **This was a qualitative study based on the methodology of phenomenology. All penultimate year medical students at University College London Medical School (n=361) were approached for this study and altogether thirteen participants were recruited, with data collected through two focus group discussions. Thematic analysis was used to generate the coding framework.

**Results: **Five key themes emerged around student attitudes to RP, which were grouped into three domains: ‘value of RP’, ‘barriers to engagement’, and ‘strategies for enabling RP’. ‘Value of RP’ centred on the themes of humanising medicine and developing empathy, developing professionalism and RP as a tool for sense-making. ‘Barriers to engagement’ centred on the purpose and tokenism of RP and in the third domain, ‘strategies for enabling RP’, the theme of student agency in RP emerged strongly.

**Conclusion: **Overall, the value of RP was not fully appreciated until students began their clinical placements. Potential strategies identified by participants for optimising engagement included student co-design and positioning RP within a broader pastoral role early in the undergraduate curriculum.

## Introduction

Reflective practice (RP) is the process by which thoughts are ‘turned back’ and analysed, with the insight gained used to shape future behaviours and practice ( Sandars, 2009). Advocated by the UK medical regulator, the General Medical Council (GMC), RP currently forms a core component of all medical students’ and doctors’ professional development in the UK and elsewhere ( GMC, 2022a; GMC, 2019). With meaningful reflection shown to benefit both patients and clinicians alike ( Wood, 2016), encouraging its adoption early in the undergraduate medical curriculum has been recommended for instilling healthy lifelong reflective practices ( Gishen & Zervos, 2018).

### Reflection and reflective practices within medical education

The concept of reflection was described by Dewey in 1933 “as a rigorous, intellectual process generating meaning and personal growth” ( Dewey, 1933). While the definition has subsequently evolved, reflection was defined as a form of “systematic enquiry to improve understanding of practice” ( Lucas, 1991) in this study. Forming a key element of Kolb’s (1984) learning cycle reflection has been shown to be a powerful tool for developing resilience and limiting the effects of burnout among clinicians ( Gishen *et al*., 2018). 

Within undergraduate medical education, various strategies are recommended for enabling engagement with RP ( GMC, 2019; Mann *et al*., 2009), including

- Written reflection: Here individuals are encouraged to consider an experience, with the aim of focusing their thoughts and feelings to create new insights that can guide their future practice ( Naber & Markley, 2017).
- Small-group work and case-based learning: This can take the form of role-playing or high-fidelity simulation training where each individual is allocated a specific role. By having the opportunity to see how they perform within a safe setting, this can enable individuals to gain multiple perspectives and advance their practice ( Khan *et al*., 2011).
- Inquiry based teaching: Here individuals are encouraged to look at a problem from multiple perspectives and create new approaches ( Gunderman & Kanter, 2009). Often used in clinical settings, appropriate role modelling, mentorship, and providing constructive feedback have all been shown to be important in inquiry-based teaching ( Spear-Ellinwood, nd).

Broadly expanded from Schon’s work on ‘reflection in- and on- practice’ ( Schon, 1983) the aim is that by adopting a range of strategies, medical students will develop an iterative way of thinking, enabling them to reflect upon their practices and improve their clinical approach ( Reflective practice, 2022).

## Reflective practice at University College London (UCL) medical school (UCLMS)

The UCL MBBS (Bachelor of Medicine, Bachelor of Surgery) curriculum consists of a six-year programme, split between ‘pre-clinical’ (Year 1–2: Foundations of Clinical Science), an integrated Bachelor of Science (Year 3), and ‘clinical’ years (Year 4–6, based in clinical placements). Adhering to the blueprint developed by the GMC (2022b), a range of RP activities have been incorporated into the ‘professionalism’ module, as part of the vertical teaching strand: Clinical and Professional Practice (CPP) ( Table 1). This draws on the pedagogical principles of spiral learning, with increasingly complex material layered and advanced throughout the course. While all students are required to participate in small group work led by trained clinical facilitators in the pre-clinical years of the MBBS Programme, other compulsory activities include the completion of marked reflective assignments across the undergraduate programme. However, participation in other forms of RP such as Schwartz Rounds (SRs) and Balint groups in the clinical years remains voluntary.

**Table 1.** Summary of mandatory and optional reflective practice learning in the University College London Bachelor of Medicine, Bachelor of Surgery (UCL MBBS) curriculum. ( https://uclms-asr.app/).

MBBS Year Group	Reflective learning opportunities
Year 1 and 2	*Mandatory:* • Small group discussion • 7–9 short portfolio reflective assignments based on early patient contact • Short written reflections on clinical encounters
Year 3 (iBSc)	No formal reflective practice
Year 4	*Mandatory:* • Two written assignments. 500–1000 words, unstructured • Reflection on Supervised Learning Events *Optional:* • Balint groups • Student psychotherapy scheme • Schwartz Round
Year 5	*Mandatory:* • Three written reflections required for end of module ‘sign off’ • Reflection on Supervised Learning Events *Optional:* • Schwartz Round
Year 6	*Mandatory:* • Reflection on Supervised Learning Events *Optional:* • Schwartz Round

### Challenges to engagement with RP

Despite RP being recognised as core to professionalism ( Sandars, 2009), this appeared to be disconcordant with medical students’ perceptions on the value of RP at UCLMS. Data gathered from the student evaluation questionnaires (SEQs) showed how many students did not appear to view RP as an important aspect of their curriculum. Furthermore, a student-led survey in 2019 also identified how many students resented having their personal reflections marked and graded ( Lalani *et al.*, 2019).

“Many of us also feel that reflection is personal, subjective and does not lend itself to grading. Receiving a low grade can be demoralising and can imply that the student has reflected ‘incorrectly’, which many students find inappropriate. I can also see how fulfilling specific grading criteria may encourage contrived writing at the expense of genuine reflection.”

Within the literature, it has been suggested that scepticism around RP is predominantly due to learning being assessment-driven among medical students ( Farmer, 2015). In addition to the inherent difficulties in assessing RP in conventional examinations, it was also identified that RP often remained largely unappreciated until students become immersed in clinical practice ( Farmer, 2015). While unclear aims and a lack of integration within the curriculum have also been suggested as potential attributing factors ( Lempp & Seale, 2004), the role of organisational and cultural factors, including the impact of ‘negative’ role modelling have been recognised to affect how medical students view reflection and subsequently develop their own coping strategies ( Sandars, 2009). However, it is also important to acknowledge the legacy of the Bawa-Garba case on RP. Following the trainee doctor’s written reflections being subpoenaed as evidence in a manslaughter case, deep insecurities continue to endure within the profession ( Dyer & Cohen, 2018; Medisauskaite *et al.*, 2021). Despite the GMC updating their guidance ( GMC, 2021), three out of four doctors acknowledged that they had reduced the amount of written reflection in their professional portfolio as a result of this case ( BMA, 2018; Furmedge, 2016).

At a time when the medical profession globally faces unprecedented pressures due to the impact of COVID-19 ( Chor *et al.*, 2021) and with medical students themselves at significant risk of stress and burnout ( Dyrbye *et al.*, 2006; Hill *et al.*, 2018), encouraging RP remains fundamental to the ‘duty of care’ of medical and clinical educators ( Hatem *et al.*, 2011). While it was acknowledged that there was a strong body of literature on reflective practice in medicine and medical education ( Aronson, 2011), studies specifically exploring the perspective of the medical student on RP remains scarce. By critically examining curricular opportunities through the student lens, the aim was to identify what RP is, the barriers to engagement, and strategies for strengthening involvement within the undergraduate MBBS Programme. Led by a senior clinical academic with a background in medical education, this study was undertaken as part of a Doctoral research project in 2018–19.

## Methods

### Ethical statement

This study was undertaken by the Principal Researcher, FG, as part of an Institution Focused Study (IFS) in Year 2 of the UCL Doctor of Education (EdD) programme. Ethical approval was gained from UCL Institute of Education Research Ethics Committee (REC). Written informed consent was obtained from participants prior to participation in this study.

### Methodology

A phenomenological approach ( Laverty, 2003) was adopted for this study, as a key aspect was to ensure that the experiences and interpretation of the reflective components in the curriculum by the medical students were explored and interpreted. Originally described by Husserl at the start of the 20 ^th^ century, phenomenology considers individuals’ lived experiences and intentionality, acknowledging that every encounter is framed by their background or situatedness in society and history ( First Philosophy, 1920–1925). Presently, many different sub-types of phenomenology are described in the literature and for this study, Hermeneutic phenomenology (HP), which explores the interpretative structures of experiences between participants, researcher and the real world, was used ( Smith, 2018).

### Participants

It was important for this study that participants had been exposed to all the reflective activities available in the curriculum and therefore, only senior Year 5 medical students (n=361) were invited to take part. Having first been signposted to this activity at a Schwartz Round, Year 5 students received an invitation to participate *via* the virtual learning environment, Moodle. If they responded affirmatively, they were then sent an information sheet outlining the aims and objectives of the study (Extended data- Participant Information Sheet) ( Gishen, 2022b).

Altogether 13 medical student participants were recruited to two focus groups (six males and seven females) and a consent form was signed prior to the focus groups being conducted (Extended data- Consent Form) ( Gishen, 2022b). Participants were identified as either unidentified female (UF) or unidentified male (UM) in the transcript to enable researchers to explore potential gender differences when discussing certain themes.

### Data collection

The method of data collection in this study was through focus group discussions. Interviews are often the favoured approach for data collection in phenomenological studies due to the difficulties in disentangling the individual account from the ‘group’ voice when conducting focus groups ( Love *et al.*, 2020). However, one of the advantages of conducting focus groups is that it can lead to more animated discussions and enrich the data compared to conducting sole interviews ( Flowers *et al.*, 2001; Stewart & Shamdasani, 2015). With previous research suggesting a general sense of apathy towards RP, it was acknowledged that engaging students on this topic through an hour long semi-structured interview would be difficult. Therefore, to ensure recruitment and enhance the discussion around RP, focus group discussions were used in this study and both researchers engaged in the literature outlining how focus groups can be adapted for phenomenological research ( Love *et al.*, 2020). While six to 12 participants are typically recommended for a focus group, in this study the number of participants in each group were kept low to enable participants the chance to talk and share their experience ( Morgan, 1997).

Both focus groups discussions were held prior to the summative exams near the end of the academic year in May 2018 to ensure that all aspects of students’ current RP curricula had been completed. The group was facilitated in a private room at the medical school by a final year UCLMS medical student, as it was recognised that students would likely feel more comfortable discussing aspects of the curriculum with a fellow peer than with a senior faculty member. The facilitator received training prior to undertaking the focus group from the principal researcher.

A pre-determined schedule was used to guide the focus group discussion around reflective practice (Extended data-Reflective Practice Focus Group Schedule) ( Gishen, 2022b). The schedule was previously piloted on a Clinical Teaching Fellow to check that the appropriate questions and timings of focus groups were feasible. This was similarly piloted in a private room at UCL medical school and no changes were made to the schedule. The focus groups lasted around 60 minutes and these were audio recorded before being transcribed using an external transcription service. All audio-recordings were subsequently destroyed following the completion of the data analysis process.

### Data analysis

Reflective thematic analysis (RTA) was used for data analysis and was undertaken by the principal researcher and the trained facilitator. Originally described by Braun & Clarke (2006), here the data were coded initially ‘line by line’ before being iteratively grouped into concepts and key themes based on the data. Both coding frameworks were then compared between the principal researcher and student facilitator to ensure congruence in how the data had been interpretated ( Table 2). 

**Table 2.** Coding framework.

**Code**	Theme
Humanizing healthcare Development of resilience and empathy Fostering supportive learning environments and student well-being Liminality; where do medical students belong?	Humanizing medicine and developing empathy
Self-development; reflexivity Developing criticality Understanding professionalism Growing as a person	Nurturing professionalism & developing criticality
Not appreciating its importance or function Lacking reflective maturity The potential role of reflective peer mentors Helping think about doing the ‘right thing’	A tool for sense making & promoting social justice
Questioning value, purpose of RP Artificiality Tokenism, ‘tick-box’ Waste of time; futility Not examined in assessments Coherence, relevance to the rest of the course	Troubling purpose and tokenism of RP
Format is wrong Appeal Palatability Branding Powerlessness Antagonism towards written assignments & Grading Mistrust for Faculty/Hidden Agenda	Student Agency in the RP Curriculum

This technique of triangulation is widely recognised in the literature as a crucial step for ensuring robustness ( Duffy, 1987). Member-checking of the preliminary data analysis with one of the participants from the focus group discussion was also undertaken to gauge whether this evaluation reflected the student voice ( Carlson, 2010). 

## Results

Five major themes emerged from this study, which were grouped into three domains: value of RP, barriers to engagement with RP, and strategies to enable RP ( Table 3). These key themes, along their relevant domains will be explored in greater depth below, with all direct quotations from participants identified as either unidentified female (UF) or unidentified male (UM) ( Gishen, 2022a).

**Table 3.** Themes for medical student attitudes to reflective practices (RP).

Domain	Themes
Values of RP	Humanizing medicine and developing empathy
	Nurturing professionalism and developing criticality
	A tool for sense making and promoting social justice
Barriers to engagement	Troubling purpose and tokenism of RP
Strategies to enable RP	Student agency in the RP curriculum

### Value of RP

It was clear that the emotional impact of working in a clinical environment was significant and for most students was the first time they had encountered challenging and sick patients. With most students acknowledging that prior to clinical placements, they had not fully appreciated the emotional impact and at times, the ‘moral injury’ ( Murray *et al*., 2018) associated with patient care, RP acted as a tool to enable the students to understand and relate to the patient experience of coping with witnessing suffering.

“Overall, I think RP has been a way to reconnect and reconfigure my relationships to patients - it allows me to see them both as people, diseases and bodies, and helps me understand how these three entities interact.” (UM)

The role of RP in humanizing medicine and developing empathy appeared to be particularly important when encountering loss, with participants describing how it made me them think about how they would behave and practice in the future when faced with such a scenario.

“The first time I saw a patient, they were crying for hours and you just don't know what to do with that, and so you have to reflect. Reflection should help us pre-think that situation – what could I do in that situation or even afterwards?”(UF)

It was also recognised that RP was a valuable for nurturing professionalism & developing criticality as an individual. This concept of RP as a learning and self-development tool featured prominently in the focus group discussion:

“[RP] is useful because it means that you get better and it’s all about improvement and providing the best service of care for your patients rather than just being complacent and continuing doing the things that you do.” (UM)
“I think I became more reflective in what I do because a) my own health reasons, b) because I have looked at the way I’ve studied and think how it is more effective, how can I make it better, so I’ve got more time to do other things.” (UF)

However, it was acknowledged by several participants that RP was not just for self-improvement but also acted as a tool for sense-making, enabling students to look out for each other and make sense of difficult situations.

“And I think because you see the patients and you see what they’re going through … how are they coping with things? And how are they not coping with things? And then also you see your friends as well, are they incurring difficulties? By learning about reflective practice…. you can help others as well.” (UF)

It was clear among the students that RP had power in helping them to understand and process the emotional burden of looking after sick patients. More importantly, it engendered empathy and camaraderie among the students, enabling them to better support each other through their shared experiences.

### Barriers to engagement with RP

While participants highlighted the value of RP, it was equally apparent that this had not been fully appreciated until they had been exposed to the clinical environment, which at UCLMS predominantly occurs from Year 4 onwards.

“…(When) you first go into a hospital, people start dying around you for the first time, you start seeing really dire circumstances and real humans suffering for the first time, and we don't get any lectures about that and we don't get enough preparation for that.” (UF)

RP was considered to lack both coherence and relevance to the undergraduate programme, with many participants not fully understanding the purpose, nor the importance, of engaging with RP especially in the pre-clinical years.

“[RP] was relatively out of the blue and quite disconnected from the rest of our teaching”. (UM)
“I thought that a lot of the times that the Medical School make us do reflective practice, and a lot of the time it’s met with despair – a bit like, oh my God, why do we have to do this?” (UF)

Engagement with written reflections and the completion of the compulsory portfolio assignments also appeared particularly problematic. These were often considered of low priority and for many, a ‘tick-box’ exercise, compared to directly examinable content in the undergraduate syllabus.

“The end goal at the end of the day for the majority of people is ‘I want to pass my final exams, I want to pass my fourth year, fifth year exams’, so then they’ll think, ‘Am I going to spend these next three hours learning about something or doing past questions or three hours writing my [reflective] essay?’”. (UF)

The low engagement and almost robotic process by which these assignments were completed was also highlighted:

“You’ve got your reflective piece, you’ve left it to the last night... You go, okay what do they want me to say? Have I said it in enough words? Have I mentioned ‘this made me feel’, or ‘on reflection I’… you’re using stock phrases...you have to have the word count.” (UF)

However, the grading of written reflective pieces was particularly contentious, and many participants felt that it took away from the essence of reflection and instead became an exercise focused on writing the *‘right thing’:*

“You learn a formula for reflecting, and you get to this spot where you’re not reflecting so much as you’re ‘performing’ reflection… you’re distanced from actually engaging your feelings – you take real events that have happened and then you create feelings around them.” (UM)

It was also highlighted that the quality or depth of reflection was open to bias between individual markers thereby reducing for many participants, the inherent value of RP.

“We were given certain grades and to me, when someone gives me a grade on my reflection, I just think it’s quite inappropriate. I don't think that people should grade my feelings or how I feel about certain things.” (UM)

While the perceived tokenistic element of RP formed one of the main barriers for student engagement, students’ insecurities about how these private reflections could be used also emerged during the focus group discussions.

“I think the implication from a lot of the way we’re fed reflective practice stuff, that there is a right way to reflect and a wrong way to reflect is problematic.” (UM)

Students appeared guarded about documenting their reflections and expressed their concerns that they could be used for ‘political’ or ‘punitive’ purposes. It was suggested that these anxieties had ‘filtered down’ from practicing clinicians, affecting their subsequent engagement with RP and in particular, written formats of RP.

### Strategies to enable RP

Participants in the focus group challenged the notion that RP was consistently and optimally used in a student-centered way in the UCLMS undergraduate curriculum. Instead, it was highlighted that using RP in a more supportive and pastoral sense, especially in the pre-clinical years, would enable engagement:

“Why can’t you be asked to reflect about things that are happening in your life as a student and your professional relationships and your relationships with your tutors or something? I think that would be much more organic than having quite a forced clinical experience, and having to force reflection on there?” (UF)

The constructive element of informally engaging with RP was also acknowledged as being important:

“So, you have a terrible experience with something, something ridiculous happens on a placement or on a ward or something and you go back and discuss it. This happened today…XYZ happened; that is in its own way a reflective practice in that small group because you and two other people over dinner or drinks or something, so it’s not necessarily like everyone doesn’t reflect.” (UM)

However, linking all of this together was the notion of having senior medical students guiding RP learning and role modelling. This was one of the key strategies that emerged during the focus group discussion for enabling engagement in RP.

“With the fourth years you get the idea that you’re guiding them as well and people like teaching, people like sharing their experience especially with someone who is going to be going down that path themselves.” (UF)

This idea of having someone relatable or a near-peer facilitating reflective experiences was considered by many of the participants as following a more natural format than those currently incorporated into the curriculum. Non-written formats of RP were also typically viewed more favourably for enabling genuine reflection compared to written options:

“The Schwartz Round was really interesting, and it was like someone else said, it was a very different style to what we normally do, and I did a Balint group and that really changed how I interacted in my medical placements.” (UM)

This may in part be explained by the concerns that students had on how their written reflections could potentially be used against them in medicolegal circumstances, as highlighted in the previous domain examining the barriers to engagement with RP.

It was clear during the focus group discussions that many of the participants felt that contextualizing how RP was used in the pre-clinical years and making it more relatable was vital for nurturing RP at an early stage. Having RP facilitated throughout the undergraduate curriculum by senior students, or near-peers, was highlighted as key to meaningfully engaging with RP.

## Discussion

By exploring medical students’ perceptions around reflective practice in the undergraduate curriculum, this study was innovative in identifying the perceived values, barriers and strategies for enabling student engagement with RP. While it was clear that the students had begun to appreciate the value of RP in the latter years, following patient interaction during their clinical placements and exposure to the human effects of illness, overall attitudes towards written formats of RP remained largely negative. However, a novel perspective on how engagement could be maximized at an early stage in medical education through the co-production of the reflective curriculum was identified in this study. Importantly, it highlighted that reframing RP in a more student-centered way, involving senior students and being more explicit about its purpose, especially in the pre-clinical years, was fundamental to addressing the barriers to engagement.

However, this element of staff-student partnership for driving meaningful changes within medical education requires a collaborative approach based on a shared understanding between clinical teachers and learners ( Bilodeau *et al*., 2019). Therefore, ensuring equity in the implementation of new curricular activities and also to ensure its ongoing relevance to students within the undergraduate programme is vital ( Parsons & Stephenson, 2005). While insight into how the reflective curricula can be shaped by this partnership is currently limited, with participants in this study echoing similar concerns to that of practicing clinicians, identifying how medical educators can continue to nurture medical students to be the caring and empathetic doctors of tomorrow remains key.

Despite some interesting insights from this study, its limitations should be recognised. While the main method of data collection, as described earlier, was using focus group discussions, both researchers engaged with the literature outlining how in-group discussions can be adapted for phenomenological studies ( Love *et al.*, 2020). This was important to ensure that the individual voice on how participants viewed the role of RP in the undergraduate curriculum was captured. Nevertheless, the data collected was from a relatively small number of students and with 13 out of a potential 361 participants recruited for this study, it is vulnerable to selection bias and may not be fully representative of the cohort. This is an inherent issue in any study using convenience sampling, with participation often dependent on interest in the studied topic and it was recognised that this may have led to certain generalizations among the participants that were not reflective of the overall student body at UCLMS. It should also be acknowledged that the data gathered was from Year 5 medical students at a single UK medical school: again, potentially limiting the transferability and validity of the findings. While Year 6 students may have had valuable insights, they were considered too close to their final (qualifying) examinations to be asked to participate. Although no difference in perceptions were demonstrated between male and female participants, it was recognised following the focus group discussion that identifying the participants through a unique ID and collecting further demographic characteristics beyond gender, such as age and ethnicity, may have provided further insight. While it was important in this study that data collection was limited to medical students that had experience across the breadth of the RP curriculum, conducting a larger study across a broader selection of medical schools and students may provide further insight on how factors such as participant age and also their progress through the programme affect their perceptions of RP.

Finally, the inherent issues associated with conducting ‘insider researcher’ by the principal researcher (FG) should be acknowledged. While being an ‘insider researcher’ ( Dwyer & Buckle, 2009) confers the advantage of positioning the researcher with a level of pre-understanding of the phenomenon being investigated, equally it is important to be mindful of how their own bias can influence the meaning-making process with the participant ( Fleming, 2018). Although the effects associated with this cannot be fully eliminated, the authors attempted to mitigate this by having a student facilitator lead the focus groups and member-checking the data analysis alongside a study participant.

The implications of this study within the sphere of medical education and, in particular, undergraduate curriculums are important. It was apparent that the ‘soft skills’ such as empathy, communication, and professionalism were underrated by many medical students compared to the ‘hard science’ that they learn. This may in part due to the latter being more straightforward to examine in conventional assessments and as medical schools tend to be a competitive and assessment-driven environment, until assessments truly test the ‘soft skills’ on a par with the hard science, this paradigm will be challenging to shift. However, engendering professional values and behaviours in our future doctors and equipping them with the ability to harness RP is vital for developing their resilience and wellbeing. 

## Conclusion

Optimising engagement in RP among medical students is crucial for encouraging its adoption in professional practice. While the benefits of RP are well recognised, this study provided a novel insight of the student perspective and the importance of co-creation and student agency to its uptake. With the demanding emotional and psychological burden being placed on healthcare professionals, ensuring that we instil good practices and empower students to engage in reflective practice from an early stage of undergraduate training is important for the retention and longevity of our future workforce.

## Abbreviations

BMA        British Medical Association

CPP          Clinical and Professional Practice

EdD          Doctor of Education

GMC        General Medical Council

HEI          Higher Education Institution

IFS           Institution Focused Study

MBBS      Bachelor of Medicine and Surgery

REC         Research Ethics Committee

RP            Reflective Practice

SEQs        Student Evaluation Questionnaires

UCL         University College London

UCLMS   University College London Medical School

## Data Availability

University College London Research Repository: Focus group transcript for Reflective Practice study. https://doi.org/10.5522/04/20285937.v1. ( Gishen, 2022a). The project contains the following underlying data: Focus group transcript. (Anonymised transcript of the focus groups). The data is available under the terms of the Creative Commons Zero "No rights reserved" data waiver (CC0 1.0 Public domain dedication). University College London Research Repository: Participant Information Sheet from IFS study. https://doi.org/10.5522/04/20285871.v1. ( Gishen, 2022b). This project contains the following extended data: Participant Information Sheet. (Survey used to collect participant information). Consent form. (Consent form given to all participants). Reflective Practice Group Schedule. (Schedule for the study). The data is available under the terms of the Creative Commons Zero "No rights reserved" data waiver (CC0 1.0 Public domain dedication).
