# Supplementary material for: Medical student perceptions of reflective practice in the undergraduate curriculum
Source: MedEdPublish (2016). 2022 Dec 8;12:53. Originally published 2022 Jul 25. [Version 3] doi: 10.12688/mep.19211.3 (PMC9926505; doi:10.12688/mep.19211.3)
Supplement: Peer review history

# Peer review: Reviewer response for version 3

**DOI:** 10.21956/mep.20855.r32820

**Published:** 2023-01-05

**Authors:** Abigail Ford Winkel

**Affiliations:** 1 Grossman School of Medicine, Department of Obstetrics and Gynecology and Institute for Innovations in Medical Education, New York University, New York, NY, USA

I agree with changing the status to approved with these edits.

Have any limitations of the research been acknowledged?

Partly

Is the work clearly and accurately presented and does it cite the current literature?

Yes

If applicable, is the statistical analysis and its interpretation appropriate?

Not applicable

Are all the source data underlying the results available to ensure full reproducibility?

Yes

Is the study design appropriate and is the work technically sound?

Yes

Are the conclusions drawn adequately supported by the results?

Yes

Are sufficient details of methods and analysis provided to allow replication by others?

Yes

Reviewer Expertise:

Narrative Medicine, Physician Well-Being, Coaching, Resilience

I confirm that I have read this submission and believe that I have an appropriate level of expertise to confirm that it is of an acceptable scientific standard.

# Peer review: Reviewer response for version 2

**DOI:** 10.21956/mep.20780.r32717

**Published:** 2022-11-28

**Authors:** Abigail Ford Winkel

**Affiliations:** 1 Grossman School of Medicine, Department of Obstetrics and Gynecology and Institute for Innovations in Medical Education, New York University, New York, NY, USA

The abstract is excellent - clearly stating the goals and methods and conclusions of the study. 

 Introduction: 

 First paragraph - it might make sense to elaborate on "benefit both patients and clinicians alike" because the issue of linking this work with the outcomes that hospitals and schools tend to value is essential to gain traction for the recommendations the authors later propose. 

 Methods: 

 Overall very well-described and justified, appropriate to the aims of the study. 

 One serious limitation is the risk of sampling by invitation during Schwarz rounds. How did you ensure you had a range of reactions to RP in the group that participated? This group may well be more engaged with RP to have agreed to participate in the study. Also, doing focus groups with only 13 total participants is probably too few. 4-6 focus groups of 6-10 participants each is generally recommended. Did the authors have evidence of saturation of themes from their analysis? 

 Results: 

 The concerns raised by students about the appropriateness of grading and the format of the RP assignments should be heeded: the current format may not be the best way to get the desired outcomes, but may indeed veer towards being a dreaded 'tick box.' What do the authors propose to do about this thread? 

 A notable omission from the list of types of engagement mentioned in the introduction is reading literature or engaging with other forms of art, followed by reflection in small groups, which has been shown across many studies to build community and allow development of nuanced perspectives on the work. This is absent from the curriculum and seems like a missed opportunity to encourage reflection as part of engagement in the professional community. 

 What do the authors mean by "pastoral sense"? Can this be clarified? 

 Discussion: 

 The authors could distill their analysis further to state a few concrete recommendations about what an improved format for RP would look like. This could be included as a box or table to highlight the importance of these take-aways to the reader.

Have any limitations of the research been acknowledged?

Partly

Is the work clearly and accurately presented and does it cite the current literature?

Yes

If applicable, is the statistical analysis and its interpretation appropriate?

Not applicable

Are all the source data underlying the results available to ensure full reproducibility?

Yes

Is the study design appropriate and is the work technically sound?

Yes

Are the conclusions drawn adequately supported by the results?

Yes

Are sufficient details of methods and analysis provided to allow replication by others?

Yes

Reviewer Expertise:

Narrative Medicine, Physician Well-Being, Coaching, Resilience

I confirm that I have read this submission and believe that I have an appropriate level of expertise to confirm that it is of an acceptable scientific standard, however I have significant reservations, as outlined above.

# Peer review: Reviewer response for version 1

**DOI:** 10.21956/mep.20581.r32582

**Published:** 2022-09-14

**Authors:** Rebecca L. Farrington

**Affiliations:** 1 Manchester Clinical Reasoning Group, School of Medical Sciences, Faculty of Medicine, Biology and Health, The University of Manchester, Manchester, UK

Although this study used a small sample size, it has been clearly designed and implemented using appropriate methods with sound analysis of qualitative data using recognised frameworks. It illustrates the range of difficulties encountered with student engagement in reflective practice that resonate across the spectrum of medical education.  There is a reference to contemporary literature which validates RP as important for the long term health, retention, and continued competence of practitioners, despite recent political and regulatory disconnects with its inappropriate use leading to mistrust of the process.

 The strategies proposed are sensible and achievable. They fit well with the stated aims and data analysis. 

 I felt I would have liked to know more about the limitations including the age range of participants and a description of how their maturity may have impacted the data, alongside the recognition that the nature of their self-selection bias included being recruited whilst attending highly structured RP often used in postgraduate practice.

Have any limitations of the research been acknowledged?

Yes

Is the work clearly and accurately presented and does it cite the current literature?

Yes

If applicable, is the statistical analysis and its interpretation appropriate?

Not applicable

Are all the source data underlying the results available to ensure full reproducibility?

Yes

Is the study design appropriate and is the work technically sound?

Yes

Are the conclusions drawn adequately supported by the results?

Yes

Are sufficient details of methods and analysis provided to allow replication by others?

Yes

Reviewer Expertise:

I am a medical educator based in primary care with an interest in self-care and the impact of affect on decision-making.

I confirm that I have read this submission and believe that I have an appropriate level of expertise to confirm that it is of an acceptable scientific standard.

# Response

**Published:** 2022-10-18

**Authors:** Rima Chakrabarti

**Affiliations:** University College London Medical School, UK

Thank you for your comments on this paper and especially for highlighting an important aspect to why perceptions of medical students may change as they progress through the programme.

 Although chronological age may not entirely reflect maturity levels, capturing the age range could have added an extra layer of information regarding the participants. While we did acknowledge, in the discussion, that the self-selecting nature of participants in this study could have limited the data collected (as indeed they do in any study), exploring this further was balanced against the length of the paper. However, we do take on-board the comments and are grateful for the review in adding to the rigour of the study.
